# The Dedicated Inflammatory Bowel Disease Nurse, If You Know Them, You Love Them: Survey of the Italian IBD Patients’ Association

**DOI:** 10.1093/crocol/otaf063

**Published:** 2025-11-11

**Authors:** Daniele Napolitano, Franco Scaldaferri, Salvo Leone, Enrica Previtali, Gionata Fiorino, Flavio Caprioli, Massimo Claudio Fantini, Simona Radice, Greta Lorenzon, Elisa Schiavoni, Nurse IGIBD Group, Debora Zaetta, Francesca Maria Onidi, Arianna Povoli, Teresa Sanità, Rita Sinatora, Giuseppe Morana, Anna Capriolo, Eva Roncen, Francesco Mercurio, Letizia Ercoli, Simonetta Grubissa, Valentina Pettinari, Alessandra Di Massimo

**Affiliations:** CEMAD—Fondazione Policlinico Gemelli IRCCS, Rome, Italy; CEMAD—Fondazione Policlinico Gemelli IRCCS, Rome, Italy; Università Cattolica del Sacro Cuore, Rome 00168, Italy; AMICI Onlus, Associazione nazionale per le Malattie Infiammatorie Croniche dell’Intestino, Milan, Italy; AMICI Onlus, Associazione nazionale per le Malattie Infiammatorie Croniche dell’Intestino, Milan, Italy; IBD Unit, Department of Gastroenterology and Digestive Endoscopy, San Camillo-Forlanini Hospital, Rome, Italy; Department of Pathophysiology and Transplantation, Università degli Studi di Milano, Milan 20135, Italy; Gastroenterology and Endoscopy Unit, Fondazione IRCCS Cà Granda, Ospedale Maggiore Policlinico, Milan 20135, Italy; Department of Medical Science and Public Health, University of Cagliari, Cagliari, Italy; Azienda Ospedaliero-Universitaria di Cagliari, Cagliari, Italy; Department of Gastroenterology & Endoscopy, IRCCS San Raffaele Hospital & Vita-Salute San Raffaele University, Milan 20132, Italy; Department of Surgery, Oncology and Gastroenterology (DiSCOG), University of Padova, Padova 35128, Italy; CEMAD—Fondazione Policlinico Gemelli IRCCS, Rome, Italy

**Keywords:** inflammatory bowel diseases, nursing, patient satisfaction, nurse–patient relations, quality of health care

## Abstract

**Introduction:**

Inflammatory bowel diseases (IBDs) are chronic conditions that negatively influence the quality of life of affected patients. IBD nurses are an essential part of the multidisciplinary team managing patients with IBD. Given the lack of studies evaluating the role of IBD nurses, this study aimed to assess the patient’s perspective regarding the role and competencies of the IBD nurse.

**Methods:**

A cross-sectional study was conducted between October and November 2024, using an online survey. The main inclusion criteria were being over 18 years old and having an established diagnosis of IBD. Subjects who fulfilled these criteria were invited to participate via email from the Italian IBD organization AMICI ITALIA. The questionnaire used for the survey, developed ad-hoc according to N-ECCO guidelines, was structured into 3 thematic sections: demographic and clinical information, patient–nurse interaction, and nursing competencies.

**Results:**

Most patients (69.7%) reported excellent relationships with their IBD nurse, emphasizing the importance of trust in disease management. Those who could identify their dedicated IBD nurse expressed higher satisfaction regarding empathy, communication, and the nurse’s influence on their treatment journey compared to those who could not identify their nurse, illustrating the positive impact the nurse had on their care. Similarly, patients who recognized their nurse demonstrated greater confidence in the nurse’s competence.

**Conclusion:**

This study emphasized the crucial role of the IBD nurse in the care journey of IBD patients. Although most patients reported an excellent relationship with their IBD nurse, many still struggle to identify their nurse. Larger studies are needed to confirm these findings further.

## Graphical abstract

**Graphical Abstract:**
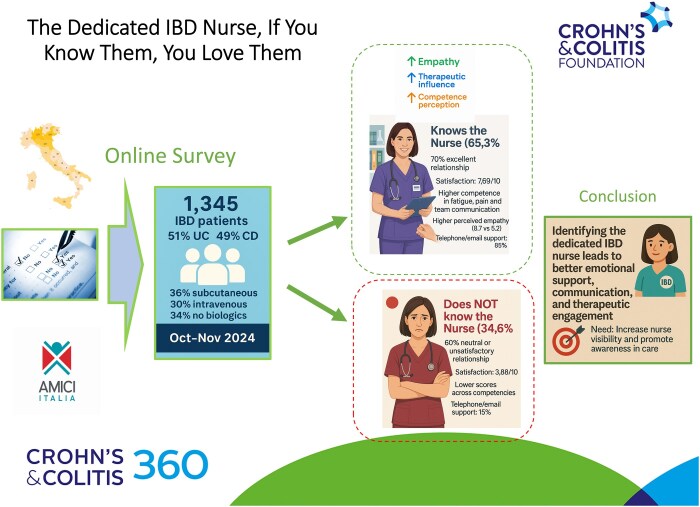

Key Messages **What is already known?**

Inflammatory bowel disease (IBD) nurses play a crucial role in patient management, providing clinical, educational, and emotional support to enhance care quality.

**What is new here?**

This study demonstrates that patients who can identify their dedicated IBD nurse report higher satisfaction, better communication, and improved perceptions of care.

**How can this study help patient care?**

Raising awareness of the IBD nurse’s role and ensuring patient access to dedicated nursing support can enhance therapeutic relationships, improve adherence to treatment, and optimize patient outcomes.

## Introduction

Inflammatory bowel disease (IBD) is characterized by chronic inflammation of the gut and clinical unpredictable course, alternating episodes of relapse and remission.^1^ The main symptoms include chronic diarrhea, abdominal pain, anemia, and fatigue. Complications may include stenosis, fistulas, and extraintestinal manifestations involving joints, skin, eyes, and liver.^2^ Diagnosis is based on a multidisciplinary approach that includes clinical, endoscopic, radiological, and histological evaluations. Treatment options range from immunosuppressive drugs to biological therapies, with a personalized approach based on the severity and localization of the disease.^1^^,^^3^ The therapeutic process is often complex and requires the involvement of a multidisciplinary team (MDT), in which each member contributes with specific expertise to patient management.^4^^,^^5^ Inflammatory bowel disease nurses (IBD nurses) are essential in managing patients with ulcerative colitis (UC) and Crohn’s disease (CD). They combine advanced clinical and relational skills to address physical, psychological, and educational needs. The second N-ECCO statement highlights the IBD nurse as a key figure in the MDT, providing care from symptom monitoring to drug management, health education, and emotional support.^6^^,^^7^ Their empathetic communication enhances quality of life.^8^ The IBD nurse’s role ranges from basic education and advocacy to advanced skills in diagnosis, treatment management, and leadership.^6^^,^^9^

Moreover, the increasing global prevalence of IBD and the complexity of available therapies, particularly the shift toward subcutaneous and oral biologics, underscore the crucial role of the IBD nurse in ensuring treatment adherence, patient education, and long-term disease management in contemporary healthcare.^10^^,^^11^ In particular, IBD nurses provide continuous support to patients, guiding them through the challenges of the disease and enhancing treatment adherence, which is essential for optimizing therapeutic outcomes.^6^^,^^12-14^ However, patients often encounter barriers in accessing care, understanding their treatment options, and coping with the psychological impact of the disease.^8^^,^^15^

The relationship between the IBD nurse and the patient is built on trust, effective communication, and empathy. Specialist nurses provide clinical, emotional, and educational support, helping to improve patient’s autonomy in managing their disease.^3^^,^^16-18^ According to the second N-ECCO consensus statements, the involvement of IBD nurses can reduce anxiety and enhance therapeutic adherence due to their ability to explain complex concepts in a clear and accessible manner and to provide an ongoing point of reference.^6^^,^^12^^,^^19^ However, despite the importance of this relationship, studies show that many patients do not fully understand the role and expertise of IBD nurses, limiting the potential impact of this figure in their care.^20^^,^^21^ Improving awareness of the IBD nurse’s role is essential to maximize the benefits of this therapeutic relationship.

Despite the growing recognition of the value of IBD nurses, patients’ perceptions of this professional figure remain under-researched.^22^ Previous studies, including the second N-ECCO consensus statements and research conducted in Italian settings, suggest that limited knowledge about the role of IBD nurses may lead to suboptimal utilization of services offered.^6^^,^^23^ Delving into patients’ opinions of IBD nurses is crucial to identifying care gaps and developing targeted educational strategies. This study aims to fill this gap, providing a basis for improving the quality of nursing care and promoting more inclusive health policies. Examining patients’ perceptions further enhances the role of IBD nurses, optimizing their contribution to the multidisciplinary care pathway and ensuring more effective and personalized support.

This study aimed to analyze and evaluate the opinion of IBD patients on the competencies of dedicated IBD nurses in Italy.

## Methods

A cross-sectional study was carried out between October 10 and November 25, 2024, as an online survey on the SurveyMonkey platform. Individuals older than 18 years old with a formal diagnosis of IBD were contacted by sending invitation emails through an Italian IBD patient organization, Associazione Nazionale per le Malattie Infiammatorie Croniche dell’Intestino, also known as AMICI ITALIA, with a purposive sampling method and completed a survey. Individuals were ineligible to participate if they were unable to give informed consent and had impaired capacity to complete the survey. No incentives were given to the participants and had provided informed consent to participate in the study by agreeing to the terms outlined at the beginning of the questionnaire. The survey was conducted second the checklist for reporting of survey studies (CROSS).^24^

### The questionnaire

The questionnaire, constructed according to N-ECCO guidelines, included questions designed to evaluate fundamental and advanced nursing competencies in IBD care. The questionnaire was developed ad hoc (Supplementary File 1) by a panel of experts based on the statements from the second N-ECCO consensus. The expert panel included 2 nurses (E.S. and D.N.) with recognized expertise in IBD care, 2 expert gastroenterologists (F.S. and G.F.), and 2 representatives from the national patient association AMICI Italia (S.L. and E.P.). It included 43 closed-ended questions categorized into 3 thematic sections.

The first section comprises demographic and clinical information. This section consists of 6 questions to collect essential demographic and clinical data. Multiple-choice questions were used to capture participants’ age, gender, region of residence, and diagnosis type (CD, UC, or indeterminate colitis). Additional questions inquired about the year of diagnosis and whether participants were receiving biological therapy, specifying subcutaneous or intravenous administration.

The second section is patient–nurse interaction. This section focused on the participants’ interaction with their care centers and dedicated IBD nurses and contained 5 questions. Binary questions (“yes” or “no”) assessed whether patients had a dedicated IBD nurse and how long they had been under care at their current center. A Likert-scale question (1-10) evaluated the relationship quality with their nurse. Another multiple-choice question explored the nature of this relationship, offering descriptors such as “excellent,” “good,” “neutral,” or “unsatisfactory.”

The third section is the nursing competencies. This section is divided into fundamental and advanced competencies. The fundamental competencies segment of the questionnaire was composed of 16 questions designed to assess essential aspects of nursing care. Participants were asked to evaluate their nurse’s performance using a 10-point Likert scale, focusing on critical skills such as empathy, effective management of biological therapies, and the ability to provide tailored educational support. These questions aimed to capture how well nurses address the emotional and psychological impact of IBD on patients, evaluate the foundational skills necessary for delivering comprehensive and compassionate care. The advanced competencies segment of the questionnaire comprised 13 questions designed to evaluate the specialized skills of dedicated IBD nurses and their role within the broader healthcare team. Most questions used a 10-point Likert scale, allowing participants to provide detailed assessments of the nurses’ expertise in several key areas. This section focused on the ability of nurses to collaborate effectively within MDT and contribute to clinical decision-making. It also addressed their competence in managing complex conditions, such as perianal disease, comorbidities, and transitional care for pediatric patients moving to adult care. Another significant aspect was evaluating their use of telematic tools to educate and support patients, aiming to enhance self-management strategies and improve adherence to prescribed therapies. Additional questions explored the nurses’ ability to address specific challenges faced by patients, such as managing incontinence and sexual health issues and providing care during pre– and post–pregnancy phases. Participants also rated the nurses’ knowledge of current regulations, technological advancements, and vaccination requirements critical to IBD management.

In addition, the segment included 3 questions examining nurses’ involvement in research activities and their ability to contribute to integrated patient care and the use of telephone or email support. Participants were asked whether their nurses were engaged in clinical trials (“yes,” “no” or “don’t know”) and rated their effectiveness in collaborating with specialists to provide holistic, multidisciplinary care and offering telephone or email support to patients.

### Statistical analysis

Descriptive statistics summarized the participants’ demographic and clinical characteristics. Continuous variables, such as age and years since diagnosis, were expressed as means and standard deviations (SD), while categorical variables were reported as absolute frequencies and percentages. Several statistical techniques were employed to explore the relationships between variables. Pearson’s correlation coefficient (*r*) was calculated to assess associations between continuous variables, such as age and years of care, and perceptions of nurse competencies. Prior to applying parametric tests, the normality of continuous variables was assessed using the Shapiro–Wilk test. A *P*-value of <.05 was considered statistically significant. Comparisons of mean values were performed using 1-way analysis of variance (ANOVA) for categorical variables with more than 2 levels, such as the type of therapy (subcutaneous, intravenous, or none). When ANOVA results indicated significant differences, post-hoc analyses, such as Tukey’s Honest Significant Difference test, were applied to identify specific group differences for comparisons between 2 groups, such as patients who could or could not identify their dedicated IBD nurse, independent sample *t*-tests were used to evaluate differences in perceived nurse competency and impact.

Associations between categorical variables, such as the identification of the dedicated IBD nurse and the type of relationship described by patients, were analyzed using the chi-squared test. Significant associations were further examined by calculating odds ratios (ORs) and 95% confidence intervals (CIs) to assess the strength of the relationships.

Missing data were handled using pairwise deletion, ensuring that all available data were included for each analysis, thereby minimizing data loss and enhancing the robustness of the findings. All statistical analyses were conducted using Jamovi software, with statistical significance set at *P* < .05.

## Results

**Figure 1.**
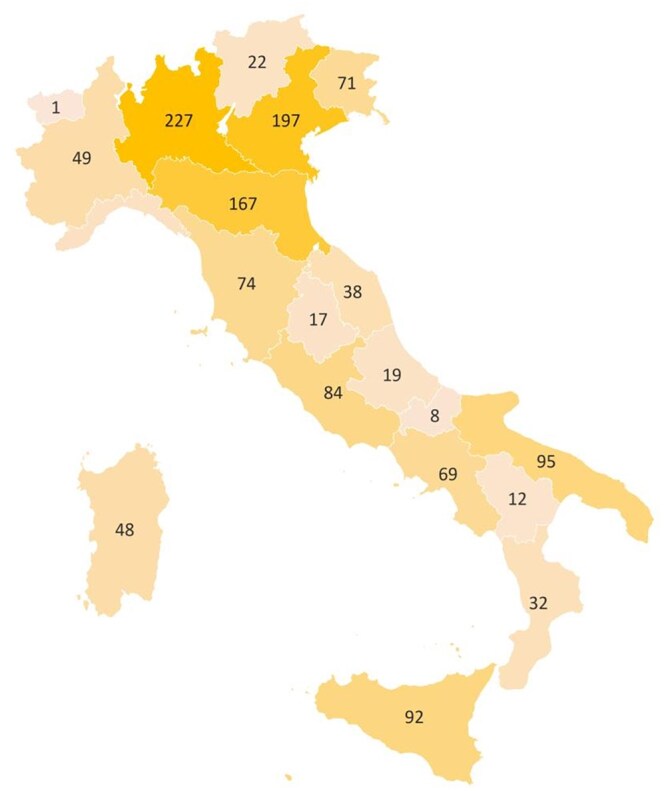
Demographic characteristics.

A total of 1512 participants responded to the survey. Of these, 120 were excluded due to incomplete responses or premature termination of the online survey. An additional 47 participants were identified as outliers (participants who provided only the lowest or highest possible scores across all items). Consequently, the final analysis included data from 1345 participants. Table 1 and Figure 1 summarizes the demographic and clinical characteristics and the patient–nurse interaction, shown in Figure 1, in the geographical distribution.

**Table 1.** The demographic and clinical characteristics and patient–nurse interaction.

Characteristic	Number (n)	Percentage (%), SD
**Age (mean)**	54	SD (15.81)
**Gender**		
**Female**	719	53.5
**Male**	626	46.5
**Diagnosis**		
**Ulcerative colitis**	685	50.9
**Crohn’s disease**	660	49.1
**Indeterminate colitis**	0	0
**Biological therapy**		
**Subcutaneous**	490	36.4
**Intravenous**	400	29.7
**No**	477	
**Followed by specialized center**	1197	89.4
**Years since diagnosis (mean)**	15.13	SD (11.32)
**Duration of care (years)**		
**Less than 1**	79	5.8
**1-3**	324	24.1
**4-6**	278	20.7
**7-10**	237	17.6
**More than 10**	418	31.1
**Identification of nurse**		
**Yes**	879	65.3
**No**	466	34.6
**Type of relationship with IBD nurse**		
**Unsatisfactory**	57	4.2
**Neutral**	327	25.2
**Good**	269	20.8
**Excellent**	647	49.7
**Influence of the dedicated IBD nurse on diagnostic/therapeutic pathway (Likert scale 1-10)**	6.4	SD (3.12)

Abbreviations: IBD, inflammatory bowel disease; SD, standard deviation.

The analysis of the relationship between patients and their dedicated IBD nurses revealed significant differences depending on whether participants could identify a dedicated IBD nurse. Participants who identified their nurse reported a mean score of 7.69 (SD = 2.28) regarding the nurse’s perceived influence on their diagnostic and therapeutic journey, reflecting a strong impact. Conversely, those who could not identify their nurse reported a significantly lower mean score of 3.88 (SD = 3.04), indicating a weaker perceived influence (*P* < .001). Regarding the type of relationship with their nurse, among participants who could identify their nurse, 69.74% described the relationship as “excellent,” feeling supported and able to rely on their nurse for any need. Additionally, 23.21% described the relationship as “good,” acknowledging its positive nature while noting room for improvement. Only 6.60% reported a “neutral” relationship, and less than 0.5% considered it “unsatisfactory.”

In contrast, among those who could not identify their nurse, 60.52% described the relationship as “neutral,” reflecting a lack of significant interaction. A smaller proportion (16.31%) rated the relationship as “good,” while 11.80% considered it “excellent.” Notably, 11.37% reported an “unsatisfactory” relationship, indicating dissatisfaction due to a perceived lack of support or attention. The chi-squared test confirmed a statistically significant association between identifying the nurse and the type of relationship described (χ^2^ = 648.67, *P* < .001).

Additionally, patients under 45 were significantly more likely to identify their dedicated IBD nurse than older patients (*P* < .001). A positive correlation (*r* = .177) was observed between recent diagnosis and nurse identification. Among those who identified as nurse, gender differences were also significant (*P* < .001). Males were most likely to describe their relationship as “excellent” (74.43%), followed by females (65.90%) and individuals who preferred not to disclose their gender (66.67%). No significant differences were observed between patients with UC and CD regarding the identification of the dedicated IBD nurse or satisfaction scores. Similarly, patients from Northern Italy showed slightly higher identification rates and satisfaction levels compared to those from Southern Italy and Islands, but these differences were not statistically significant.

### Fundamental skills

**Figure 2.**
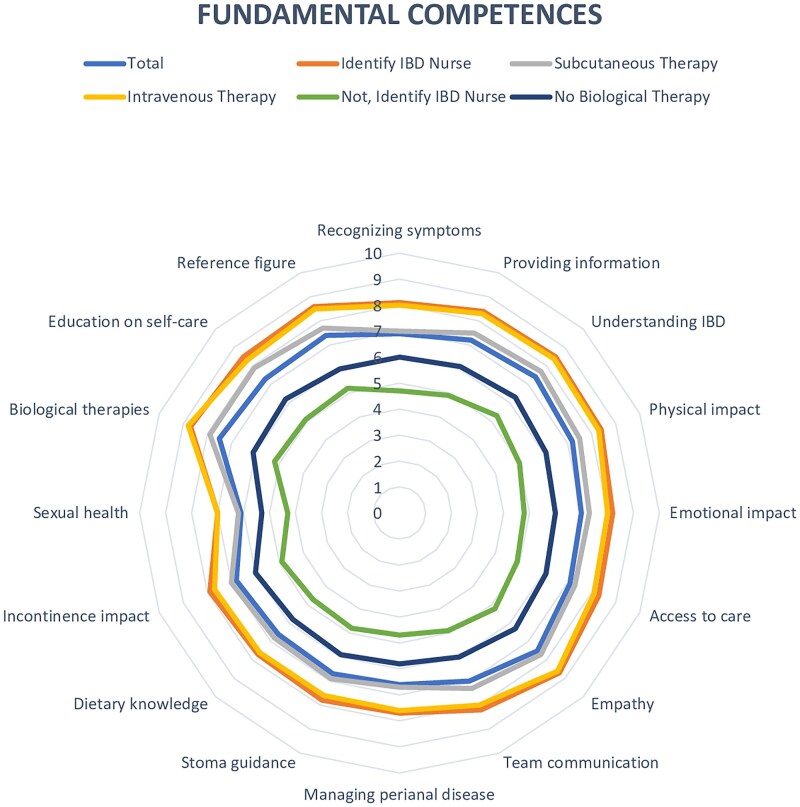
Fundamental competences.

The analysis (Table 2) showed that patients who were able to identify their dedicated IBD nurse reported significantly higher scores in several key domains, including competence in providing information (8.4 ± 1.8 vs. 4.9 ± 3.1; *p* = 0.001), awareness of the physical impact of the disease (8.4 ± 1.8 vs. 4.9 ± 3.1; *p* = 0.0001), ability to establish an empathetic relationship (8.7 ± 1.7 vs. 5.2 ± 3.1; *p* = 0.001), and competence in managing biological therapies (8.7 ± 1.7 vs. 5.2 ± 3.1; *p* = 0.001). Across all other domains, although not statistically significant, a consistent trend toward higher scores was observed among patients who recognised their nurse. No statistically significant differences were found according to therapy type, although descriptively higher scores were observed among patients receiving intravenous therapies (Figure 2)

**Table 2:** Patient’s opinion of Fundamental Competence IBD Nurse

Questiona		Total Mean ± SD	Identifies Nurse Mean ± SD	*P*-value	Not, Identify IBD nurse Mean ± SD	Subcutaneous Therapy Mean ± SD	Intravenous Therapy Mean ± SD	No Biological Therapy Mean ± SD	*P*-value
**Q1**	**How competent is your dedicated IBD nurse in recognizing symptoms of fatigue and suggesting helpful adjustments?**	6.9 ± 2.9	8.1 ± 2.0	ns	4.7 ± 3.2	7.0 ± 2.8	8.0 ± 2.4	6.0 ± 3.2	ns
**Q2**	**How do you rate your dedicated nurse’s competence in providing information about your condition?**	7.2 ± 2.9	8.4 ± 1.8	0.001	4.9 ± 3.1	7.5 ± 2.6	8.3 ± 2.3	6.1 ± 3.2	ns
**Q3**	**How well does your dedicated IBD nurse understand the differences between diagnoses and therapeutic pathways for IBD?**	7.4 ± 2.8	8.5 ± 1.7	ns	5.3 ± 3.1	7.7 ± 2.5	8.4 ± 2.1	6.3 ± 3.1	ns
**Q4**	**How competent and aware is your dedicated IBD nurse of the physical impact that your disease has on you?**	7.2 ± 2.8	8.4 ± 1.8	0.0001	4.9 ± 3.1	7.5 ± 2.5	8.3 ± 2.2	6.1 ± 3.2	ns
**Q5**	**How competent and aware is your dedicated IBD nurse of the emotional and psychological impact that your disease has on you?**	7.0 ± 2.9	8.2 ± 2.0	ns	4.8 ± 3.0	7.3 ± 2.6	8.0 ± 2.3	6.0 ± 3.2	ns
**Q6**	**How competent is your dedicated IBD nurse in identifying needs and ensuring appropriate access to the best care for patients?**	7.1 ± 2.8	8.3 ± 1.8	ns	4.9 ± 3.0	7.3 ± 2.5	8.1 ± 2.3	6.1 ± 3.1	ns
**Q7**	**How capable is your dedicated IBD nurse in establishing an empathetic relationship with the patient?**	7.5 ± 2.8	8.7 ± 1.7	0.001	5.2 ± 3.1	7.7 ± 2.6	8.6 ± 2.1	6.3 ± 3.1	ns
**Q8**	**How capable is your dedicated IBD nurse in supporting communication with the multidisciplinary team?**	7.0 ± 2.8	8.2 ± 1.9	ns	4.9 ± 3.0	7.3 ± 2.6	8.0 ± 2.3	6.0 ± 3.1	ns
**Q9**	**How competent is your dedicated IBD nurse in managing perianal disease?**	6.6 ± 2.8	7.7 ± 2.1	ns	4.7 ± 3.0	6.7 ± 2.7	7.6 ± 2.5	5.8 ± 3.0	ns
**Q10**	**How capable is your dedicated IBD nurse in managing a stoma or providing guidance on specialists to consult?**	6.7 ± 2.8	7.8 ± 2.1	ns	4.8 ± 3.0	6.9 ± 2.6	7.6 ± 2.4	5.9 ± 3.1	ns
**Q11**	**How knowledgeable is your dedicated IBD nurse about the dietary issues related to your condition?**	6.6 ± 2.8	7.7 ± 2.1	ns	4.7 ± 3.0	6.8 ± 2.6	7.6 ± 2.5	5.8 ± 3.0	ns
**Q12**	**How competent is your dedicated IBD nurse regarding the impact of incontinence on patients' quality of life?**	6.8 ± 2.8	7.9 ± 2.0	ns	4.9 ± 3.0	7.0 ± 2.6	7.7 ± 2.5	6.0 ± 3.1	ns
**Q13**	**How competent is your dedicated IBD nurse in sexual health issues related to IBD, providing necessary suggestions?**	6.1 ± 2.9	7.0 ± 2.4	ns	4.3 ± 2.9	6.2 ± 2.7	7.0 ± 2.8	5.3 ± 3.0	ns
**Q14**	**How competent is your dedicated IBD nurse in managing biological therapies?**	7.5 ± 2.8	8.7 ± 1.7	0.001	5.2 ± 3.1	7.9 ± 2.4	8.8 ± 1.9	6.0 ± 3.1	ns
**Q15**	**How does your dedicated IBD nurse provide education on self-administration of subcutaneous medications to patients?**	7.3 ± 2.9	8.5 ± 1.9		5.1 ± 3.1	7.9 ± 2.6	8.3 ± 2.2	6.2 ± 3.2	
**Q16**	**How do you perceive your IBD nurse as a reference figure for the patients in your clinic?**	7.4 ± 2.9	8.6 ± 1.8		5.2 ± 3.2	7.7 ± 2.7	8.5 ± 2.3	6.0 ± 3.0	

### Advanced skills

The last analysis of the 3rd section, in advanced skills, is described in Tables 3 and 4. The study revealed significant differences in patient perceptions based on their ability to identify their dedicated IBD nurse and the type of therapy they received. Patients who could locate their nurse reported higher satisfaction, particularly in empathy and communication, with a mean score of 8.7 ± 1.7, compared to 5.2 ± 3.1 for those who could not, yielding a *P*-value of .001. The perceived impact of the nurse on their diagnostic and therapeutic journey was also higher, with a mean score of 7.69 ± 2.28 for those who identified their nurse, in contrast to 3.88 ± 3.04 for those who could not, demonstrating a highly significant difference (*P* = .0001).

**Table 3.** Patient’s opinion of advanced competence IBD nurse.

Question		Total mean ± SD	Identifies nurse mean ± SD	Not, identify IBD nurse mean ± SD	*P*	Subcutaneous therapy mean ± SD	Intravenous therapy mean ± SD	No biological therapy mean ± SD	*P*
**Q1**	How competent is your dedicated IBD nurse in recognizing symptoms of fatigue and suggesting helpful adjustments?	6.82 ± 2.84	7.93 ± 2.03	4.75 ± 2.99	**0.001**	6.99 ± 2.62	7.93 ± 2.36	5.78 ± 3.03	**ns**
**Q2**	How do you think your dedicated IBD nurse can contribute to the multidisciplinary team and clinical discussions about patients?	7.06 ± 2.77	8.07 ± 1.98	5.17 ± 3.04	**0.0001**	7.17 ± 2.64	8.06 ± 2.22	6.17 ± 3.00	**ns**
**Q3**	How well does your dedicated IBD nurse educate patients to live with the disease, also using informational materials?	6.10 ± 3.02	7.11 ± 2.54	4.20 ± 2.94	ns	6.26 ± 2.85	6.98 ± 2.95	5.23 ± 3.02	ns
**Q4**	How does your dedicated IBD nurse provide health education for the patient’s caregiver?	5.96 ± 3.05	6.98 ± 2.60	4.03 ± 2.90	ns	6.12 ± 2.88	6.83 ± 2.98	5.10 ± 3.06	ns
**Q5**	How knowledgeable is your dedicated IBD nurse about technological innovations useful in managing IBD conditions?	6.46 ± 2.94	7.51 ± 2.29	4.46 ± 3.00	**0.02**	6.66 ± 2.76	7.35 ± 2.62	5.54 ± 3.10	**ns**
**Q6**	How competent is your dedicated IBD nurse regarding current regulations and supporting patients in safeguarding their rights?	6.13 ± 3.00	7.16 ± 2.49	4.18 ± 2.91	ns	6.26 ± 2.82	7.09 ± 2.85	5.24 ± 3.04	ns
**Q7**	How competent is your dedicated IBD nurse in managing pre and post-pregnancy care for female patients?	6.14 ± 2.94	7.17 ± 2.40	4.21 ± 2.91	ns	6.23 ± 2.81	7.14 ± 2.69	5.26 ± 3.01	ns
**Q8**	How capable is your dedicated IBD nurse in informing patients about travel or healthcare needs away from home?	6.27 ± 2.95	7.30 ± 2.39	4.32 ± 2.94	ns	6.45 ± 2.75	7.13 ± 2.78	5.40 ± 3.05	ns
**Q9**	How knowledgeable is your dedicated IBD nurse about the main vaccination needs of patients and provides related information?	6.38 ± 2.97	7.43 ± 2.39	4.41 ± 2.94	**0.02**	6.60 ± 2.80	7.28 ± 2.76	5.44 ± 3.03	**ns**
**Q10**	How well does your dedicated IBD nurse recognize comorbidities and extra-intestinal manifestations related to IBD?	6.37 ± 2.94	7.39 ± 2.38	4.43 ± 2.93	ns	6.47 ± 2.77	7.29 ± 2.75	5.52 ± 3.02	ns
**Q11**	How skilled and competent is your dedicated IBD nurse in research?	6.05 ± 2.93	7.04 ± 2.45	4.18 ± 2.86	ns	6.13 ± 2.75	6.95 ± 2.80	5.26 ± 2.99	ns
**Q12**	How do you think your dedicated IBD nurse can collaborate with colleagues from other specialties to provide multidisciplinary care?	6.70 ± 2.89	7.80 ± 2.21	4.63 ± 2.89	**0.0001**	6.86 ± 2.68	7.78 ± 2.55	5.69 ± 3.02	**ns**
**Q13**	How competent is your dedicated IBD nurse in pain management?	6.71 ± 2.85	7.77 ± 2.15	4.71 ± 2.95	**0.001**	6.86 ± 2.66	7.79 ± 2.42	5.70 ± 3.02	**ns**

Abbreviations: IBD, inflammatory bowel disease; SD, standard deviation. Bold values are statistically significant

**Table 4.** Patient’s opinion of advanced competence IBD nurse.

Question	Response	Total (%)	Identifies IBD nurse (%)	Not, identifies IBD nurse (%)	*P*	Subcutaneous therapy (%)	Intravenous therapy (%)	No biological therapy (%)	*P*
**Does your IBD nurse participate in conferences and courses in the field of IBD?**	Yes	317 (23.57)	295 (93.06)	22 (6.94)	**0.001**	23 (7.26)	17 (5.36)	22 (6.94)	**ns**
	No	19 (1.41)	7 (36.84)	12 (63.16)	ns	8 (42.11)	5 (26.32)	6 (31.58)	ns
	I don’t know	1009 (75.02)	577 (57.19)	432 (42.81)	ns	357 (35.38)	247 (24.48)	426 (42.22)	ns
**Does your dedicated IBD nurse provide telephone or email support services to patients?**	Yes	713 (53.01)	603 (84.57)	110 (15.43)	**0.001**	433 (60.73)	223 (31.28)	57 (7.99)	**ns**
	No	632 (46.99)	403 (63.77)	229 (36.23)	ns	328 (51.9)	147 (23.26)	157 (24.84)	ns
**Does your dedicated IBD nurse conduct clinical studies?**	Yes	28 (2.64)	123 (439.29)	17 (60.71)	ns	54 (192.86)	58 (207.14)	28 (100.0)	ns
	No	23 (2.17)	36 (156.52)	27 (117.39)	ns	23 (100.0)	17 (73.91)	23 (100.0)	ns
	I don’t know	1009 (95.19)	577 (57.19)	432 (42.81)	ns	357 (35.38)	247 (24.48)	426 (42.22)	ns

Abbreviation: IBD, inflammatory bowel disease. Bold values are statistically significant

**Figure 3.**
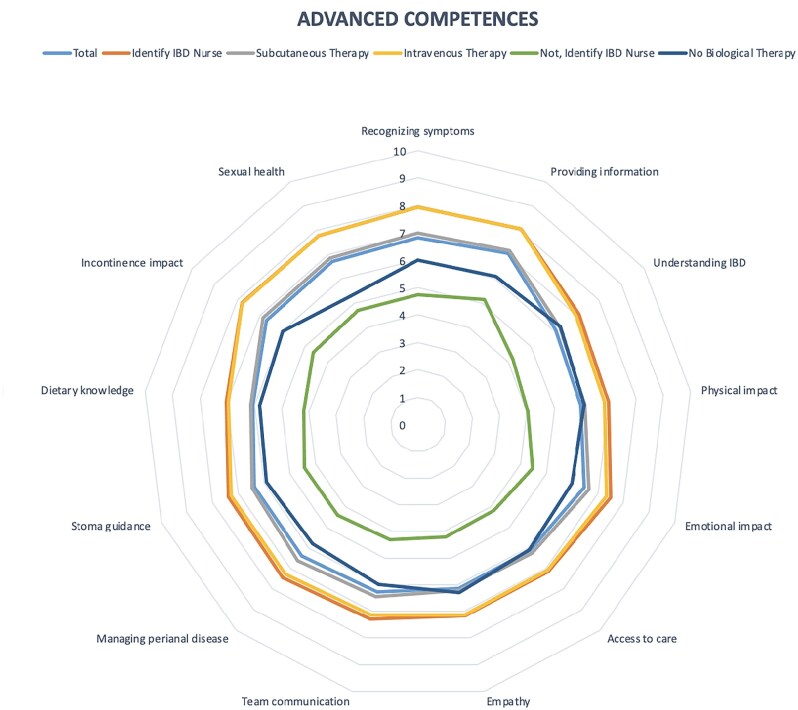
Advanced competences.

Patients who could identify their nurse also reported greater confidence in their competence, especially in disease management and information provision, with a mean score of 8.4 ± 1.8, compared to 4.9 ± 3.1 for those unable to do so (*P* = .0001). Furthermore, patients receiving subcutaneous therapy rated their nurse’s competence and impact on care significantly higher than those not undergoing subcutaneous treatment, achieving a mean score of 8.6 ± 2.1 for competence in managing biological therapies (vs. 6.0 ± 3.2) and a mean score of 7.9 ± 2.4 for impact on care (versus 6.0 ± 3.1), both with statistically significant *P*-values of .02 and .01, respectively (Figure 3).

The analysis revealed significant associations: patients who identified their dedicated IBD nurse were more likely to perceive them as participating in conferences and courses in the field of IBD (*P* < .001), while the type of biological therapy influenced the provision of support services, including telephone or email support (*P* < .001) (Table 4).

## Discussion

**Figure 4.**
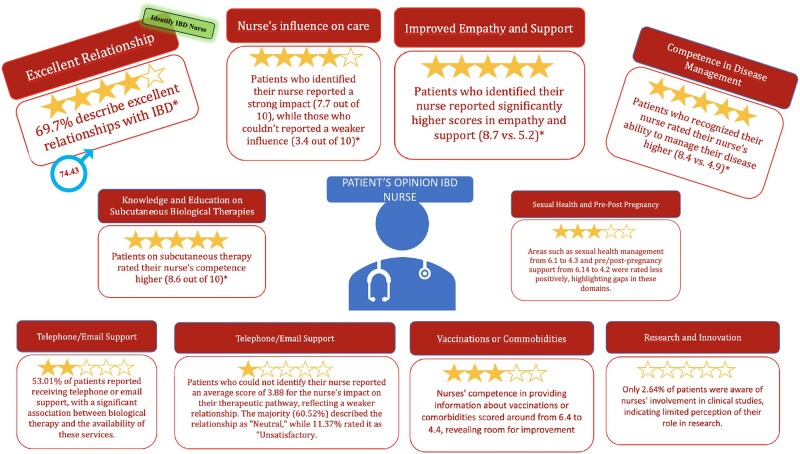
Opinion of IBD patients.

This study aimed to analyze and assess the perspectives of patients with IBD regarding the competencies of dedicated IBD nurses in Italy. To achieve this, a panel of experts developed a questionnaire based on the second N-ECCO statement, the key ECCO document outlining the competencies of dedicated IBD nurses.^6^ The findings indicate that patients’ recognition of dedicated IBD nurses is strongly associated with greater satisfaction with care pathways and a more favorable evaluation of nursing competencies. This association is particularly evident among patients receiving biological therapy. Additionally, the study identifies specific areas requiring further attention, including sexual health education and caregiver involvement, highlighting the need for targeted interventions and improvements (Figure 4). The ability of IBD patients to recognize their dedicated nurse was consistently linked to higher scores across all assessed variables. A significant proportion of patients reported recognizing their dedicated IBD nurse, which is particularly relevant as it highlights the role of key healthcare professionals in managing the disease. This recognition was especially evident among those undergoing biological therapy.

Additionally, although a slight trend toward higher nurse identification and satisfaction was observed among patients from Northern Italy compared to Southern Italy and Islands, these differences did not reach statistical significance. Future studies should investigate potential regional disparities in IBD nursing care across Italy. Conversely, patients who could not recognize their dedicated IBD nurse reported significantly lower average scores.

Nevertheless, many of these patients still rated their relationship with the nurse as “excellent.” A notable finding is that most patients unable to recognize a dedicated nurse were male. Additionally, a significant proportion of those who identified a dedicated IBD nurse as a reference figure were male and under 45.

Furthermore, a strong correlation emerged between a recent diagnosis and the ability to recognize the IBD nurse as a key point of reference. These findings align with existing literature, highlighting IBD nurses’ increasingly central role within IBD units.^25^ Specifically, in first-level IBD centers, a dedicated IBD nurse is well established, playing a pivotal role in the clinical and diagnostic pathway of patients.^26^ Similarly, no significant differences were observed between patients with CD and those with UC regarding their ability to recognize their dedicated nurse or satisfaction with nursing competencies.

The rising incidence of IBD and the introduction of novel biological therapies as first-line treatments have led to increased reliance on IBD centers from the onset of symptoms.^27^^,^^28^ This trend is reflected in the greater frequency of patient interactions with dedicated IBD nurses at these specialized centers.^29^ Notably, self-care education received high ratings, particularly among patients undergoing subcutaneous biological therapy, likely due to the direct benefits of education in managing their treatment independently.^11^^,^^30^^,^^31^ Patients receiving intravenous biological therapy reported the highest frequency and duration of interactions with dedicated nurses. This prolonged contact facilitates the development of an empathetic and trusting relationship with these professionals, often leading patients to consider them key figures in managing their healthcare. The questionnaire responses confirm this trend.

However, recent changes in treatment protocols, with the transition of some patients from intravenous to subcutaneous formulations of biologic therapies, have reduced the frequency of contact with dedicated IBD nurses. Despite this, these patients have retained the benefits gained from their previous experience with intravenous treatment.^32^ On the other hand, the area where the lowest levels of knowledge were recorded was sexual health education. The recognition of nursing competencies in this field was restricted across all categories of interviewed patients. Further investigation is needed to determine whether this gap stems from a lack of knowledge among patients and healthcare providers or if it reflects persistent discomfort in discussing the topic. Sexual and reproductive health in individuals with IBD has been the focus of increasing research in recent years, highlighting growing awareness of this critical aspect of patient well-being.^33^^,^^34^ The rise in early-onset diagnoses and the expansion of treatment options for disease management have further contributed to heightened interest in this area.^35^ While the questionnaire data suggest a perceived deficiency in nursing competence related to sexual health education, in reality, dedicated IBD nurses can play a crucial role in addressing these concerns and providing essential support.^36^

Patients undergoing biological therapy, particularly those receiving intravenous treatment, reported a higher recognition of dedicated IBD nurses’ competencies than patients not on biological treatment. This observation aligns with existing literature, highlighting IBD nurses’ ability to establish empathic relationships with patients.^37^ Notably, patients who recognize the role of dedicated IBD nurses also attribute an in-depth understanding of the disease and its management and an enhanced capacity to identify patients’ needs. This area received particularly positive ratings. These findings reinforce the nursing profession’s well-documented relational and educational strengths, which have long been considered fundamental aspects of patient-centered care.^38^ The ability of dedicated IBD nurses to engage in research was also highly rated by patients undergoing biological therapy, whether intravenous or subcutaneous. Conversely, patients not receiving biological treatment, regardless of whether they recognized the nurse’s role, were less likely to acknowledge their expertise in this domain.^39^

Regarding advanced competencies, patients who recognized the dedicated nurse provided significantly higher ratings than those who did not. In this context, the highest scores were attributed to nurses’ interpersonal skills and patient support. In particular, nurses’ ability to identify symptoms of fatigue was highly acknowledged, aligning with recent literature that underscores the importance of fatigue assessment in IBD, particularly from a nursing perspective. Another critical aspect concerns the recognition and management of pain in IBD patients.^40^ While healthcare professionals often underappreciate pain compared to patients’ assessments, dedicated IBD nurses were recognized for their capacity to identify pain and provide appropriate recommendations interestingly, patients receiving biological therapy reported significantly higher scores in this domain compared to those not on therapy, further supporting the trend that patients undergoing biological treatment exhibit greater awareness and appreciation of the role of dedicated IBD nurses.^41^ Nurses’ ability to collaborate with other healthcare professionals to ensure MDT was a well-recognized competency. Notably, the highest-rated competence in this category was their participation in interdisciplinary meetings.^42^ Nurses are increasingly involved in these meetings due to their unique position as patient advocates, being the healthcare providers in closest contact with patients and often those gathering crucial patient-reported information.^36^ Another noteworthy area of competence is nurses’ involvement in clinical research. While patients who recognized the role of dedicated IBD nurses acknowledged their contribution to the study, a substantial proportion of respondents reported uncertainty regarding whether nurses were actively involved in research protocols.^43^ Literature has provided several examples of how nurses participating in clinical trials significantly contribute to their execution and quality.^44^

Another competence strongly attributed to dedicated IBD nurses is their knowledge of technological innovations that support patients. The most frequently cited technological support included telephone helplines and dedicated email services.^25^ Notably, patients who did not recognize the professional role of dedicated IBD nurses assumed that nurses played no role in managing these support lines. In contrast, patients undergoing subcutaneous biological therapy showed the highest recognition of their technological contributions. Literature further supports the importance of a dedicated nurse in managing telephone- and telemedicine-based helplines, demonstrating their effectiveness in reducing hospitalizations and preventing disease exacerbations.^45^ Patients who recognized the role of dedicated IBD nurses also associated them with a greater commitment to patient education regarding disease management.^25^ However, the area that received the lowest ratings was caregiver education. Although nurses are widely acknowledged for their expertise in educating patients, this competence does not seem to extend to caregivers. Further investigation is needed to determine whether this gap results from limited interaction with caregivers or reduced attention to their educational needs. While this topic has not been extensively explored in the context of IBD, existing literature suggests that strong nurse–caregiver relationships, alongside effective nurse–patient interactions, can significantly improve care outcomes.^46^

This study has some exciting strengths. The starting sample size is substantial, providing a good foundation for the study. Even after exclusions, the final sample of 1345 is still robust. The study reports numerous statistically significant findings, suggesting a strong relationship between nurse identification and positive patient perceptions.

Furthermore, the study addresses the crucial aspect of the patient–nurse relationship, which is often overlooked in research. It delves into specific aspects of the patient–nurse interaction, such as empathy, communication, competence, and impact on the treatment journey. This granular approach provides valuable insights. Finally, the study acknowledges and analyzes the influence of factors like patient age, gender, type of therapy, and time since diagnosis.

Points of weakness can be found in the fact that the data relies heavily on self-reported patient perceptions, which can be subjective and influenced by various factors. While valuable, future research could supplement this with more objective measures. The analysis does not consider the severity of the patients’ IBD when completing the questionnaire. Another limitation is that the severity of the patients’ IBD was not assessed. Given that disease activity could significantly influence patients’ experiences and perceptions of care, the absence of this information limits the interpretation of our findings. Future studies should include objective measures of disease severity, such as clinical disease activity scores, to better understand the relationship between disease status and the patient–nurse interaction. This could influence their experiences and perceptions. Moreover, while the study demonstrates strong associations, it does not necessarily prove causation. For example, patients who are more engaged with their care might be more likely to identify their nurse and have better outcomes. The relationship may not be solely because of nurse identification.

Future research may include supplementing the quantitative data with qualitative research (e.g., interviews, focus groups) to explore the nuances of the patient–nurse relationship and understand why patients who identify their nurse have more positive perceptions. The study can gain strength by incorporating objective measures of patient outcomes (e.g., disease activity scores, hospitalizations, medication adherence) to assess the impact of the patient–nurse relationship on actual health outcomes. It would also be interesting to investigate further the large group of patients who reported a “neutral” relationship with their nurse, understanding which factors contributed to this neutrality and what could be done to improve these relationships. Investigating factors contributing to this neutrality and identifying potential interventions to foster more positive connections would be highly beneficial. Expanding the research scope to a European level would allow for a cross-cultural comparison of patient perceptions and identify potential variations in the patient–nurse relationship across diverse healthcare systems and cultural contexts. This would provide valuable insights into best practices and areas for improvement on a broader scale. Increasing patient awareness of the specialized role of dedicated IBD nurses is crucial, especially considering the strong correlation between patient identification with their nurse and more positive perceptions of care. A multi–faceted approach is needed to effectively educate patients about the dedicated IBD nurses’ expertise and their comprehensive support. Developing and implementing educational programs for IBD patients could highlight IBD nurses’ unique skills and knowledge. Delivering these programs through various channels, such as online webinars, in-person group sessions, or individual consultations, could maximize reach and accessibility. Launching comprehensive informational campaigns could raise broader awareness of the dedicated IBD nurse’s role within the healthcare team. These campaigns could utilize diverse platforms, including hospital websites, social media, patient advocacy group newsletters, and printed materials distributed in clinics and hospitals. The content should clearly articulate how dedicated IBD nurses can assist patients, emphasizing their expertise in areas like managing flares, providing emotional support, coordinating care, and connecting patients with relevant resources. Sharing patient testimonials and success stories could further personalize the message and demonstrate the tangible benefits of engaging with a dedicated IBD nurse. Beyond raising awareness, empowering patients to actively utilize the resources available, including dedicated IBD nurse support, is essential. This involves communicating the process from a dedicated IBD nurse contact to a visit schedule, thus ensuring easy access to their services. Furthermore, encouraging open communication between patients and dedicated IBD nurses can foster a strong therapeutic relationship, making patients feel comfortable seeking guidance and support whenever needed.

## Conclusions

This study strongly emphasizes the critical role of the IBD nurse in patient care. The findings demonstrate that patients who can identify their dedicated IBD nurse report significantly more positive experiences across multiple dimensions. They perceive their nurses as more empathetic, communicative, and competent and feel they positively impact their diagnostic and therapeutic journey. This highlights the importance of ensuring that patients have access to and can quickly identify their dedicated IBD nurse. The study provides compelling evidence to support investment in IBD nurse staffing, training, and strategies to foster strong patient–nurse relationships. It underscores the IBD nurse’s role not merely as a provider of technical care but as a crucial source of support, education, and advocacy for patients with a chronic condition.

## Supplementary Material

otaf063_Supplementary_Data

## Data Availability

Data are not publicly available.

## Brief Report

This study highlights the crucial role of inflammatory bowel disease (IBD) nurses in patient care. Patients who identify their dedicated nurse report greater satisfaction, improved communication, and stronger therapeutic relationships. Enhancing the awareness and accessibility of IBD nurses could optimize patient outcomes and support.
