# Correction to: The Dedicated Inflammatory Bowel Disease Nurse, If You Know Them, You Love Them: Survey of the Italian IBD Patients’ Association

**DOI:** 10.1093/crocol/otag089

**Published:** 2026-08-11

This is a correction to: Daniele Napolitano, Franco Scaldaferri, Salvo Leone, Enrica Previtali, Gionata Fiorino, Flavio Caprioli, Massimo Claudio Fantini, Simona Radice, Greta Lorenzon, Elisa Schiavoni, Nurse IGIBD Group, The Dedicated Inflammatory Bowel Disease Nurse, If You Know Them, You Love Them: Survey of the Italian IBD Patients’ Association, *Crohn’s & Colitis 360*, Volume 7, Issue 4, October 2025, otaf063, https://doi.org/10.1093/crocol/otaf063

In the originally published version of this manuscript, Table 3 and the corresponding results were mistakenly duplicated and reported as Table 2.

**Table 2:** Patient’s opinion of Fundamental Competence IBD Nurse

Question		Total Mean ± SD	Identifies Nurse Mean ± SD	*P*-value	Not, Identify IBD nurse Mean ± SD	Subcutaneous Therapy Mean ± SD	Intravenous Therapy Mean ± SD	No Biological Therapy Mean ± SD	*P*-value
**Q1**	**How competent is your dedicated IBD nurse in recognizing symptoms of fatigue and suggesting helpful adjustments?**	6.9 ± 2.9	8.1 ± 2.0	ns	4.7 ± 3.2	7.0 ± 2.8	8.0 ± 2.4	6.0 ± 3.2	ns
**Q2**	**How do you rate your dedicated nurse’s competence in providing information about your condition?**	7.2 ± 2.9	8.4 ± 1.8	0.001	4.9 ± 3.1	7.5 ± 2.6	8.3 ± 2.3	6.1 ± 3.2	ns
**Q3**	**How well does your dedicated IBD nurse understand the differences between diagnoses and therapeutic pathways for IBD?**	7.4 ± 2.8	8.5 ± 1.7	ns	5.3 ± 3.1	7.7 ± 2.5	8.4 ± 2.1	6.3 ± 3.1	ns
**Q4**	**How competent and aware is your dedicated IBD nurse of the physical impact that your disease has on you?**	7.2 ± 2.8	8.4 ± 1.8	0.0001	4.9 ± 3.1	7.5 ± 2.5	8.3 ± 2.2	6.1 ± 3.2	ns
**Q5**	**How competent and aware is your dedicated IBD nurse of the emotional and psychological impact that your disease has on you?**	7.0 ± 2.9	8.2 ± 2.0	ns	4.8 ± 3.0	7.3 ± 2.6	8.0 ± 2.3	6.0 ± 3.2	ns
**Q6**	**How competent is your dedicated IBD nurse in identifying needs and ensuring appropriate access to the best care for patients?**	7.1 ± 2.8	8.3 ± 1.8	ns	4.9 ± 3.0	7.3 ± 2.5	8.1 ± 2.3	6.1 ± 3.1	ns
**Q7**	**How capable is your dedicated IBD nurse in establishing an empathetic relationship with the patient?**	7.5 ± 2.8	8.7 ± 1.7	0.001	5.2 ± 3.1	7.7 ± 2.6	8.6 ± 2.1	6.3 ± 3.1	ns
**Q8**	**How capable is your dedicated IBD nurse in supporting communication with the multidisciplinary team?**	7.0 ± 2.8	8.2 ± 1.9	ns	4.9 ± 3.0	7.3 ± 2.6	8.0 ± 2.3	6.0 ± 3.1	ns
**Q9**	**How competent is your dedicated IBD nurse in managing perianal disease?**	6.6 ± 2.8	7.7 ± 2.1	ns	4.7 ± 3.0	6.7 ± 2.7	7.6 ± 2.5	5.8 ± 3.0	ns
**Q10**	**How capable is your dedicated IBD nurse in managing a stoma or providing guidance on specialists to consult?**	6.7 ± 2.8	7.8 ± 2.1	ns	4.8 ± 3.0	6.9 ± 2.6	7.6 ± 2.4	5.9 ± 3.1	ns
**Q11**	**How knowledgeable is your dedicated IBD nurse about the dietary issues related to your condition?**	6.6 ± 2.8	7.7 ± 2.1	ns	4.7 ± 3.0	6.8 ± 2.6	7.6 ± 2.5	5.8 ± 3.0	ns
**Q12**	**How competent is your dedicated IBD nurse regarding the impact of incontinence on patients' quality of life?**	6.8 ± 2.8	7.9 ± 2.0	ns	4.9 ± 3.0	7.0 ± 2.6	7.7 ± 2.5	6.0 ± 3.1	ns
**Q13**	**How competent is your dedicated IBD nurse in sexual health issues related to IBD, providing necessary suggestions?**	6.1 ± 2.9	7.0 ± 2.4	ns	4.3 ± 2.9	6.2 ± 2.7	7.0 ± 2.8	5.3 ± 3.0	ns
**Q14**	**How competent is your dedicated IBD nurse in managing biological therapies?**	7.5 ± 2.8	8.7 ± 1.7	0.001	5.2 ± 3.1	7.9 ± 2.4	8.8 ± 1.9	6.0 ± 3.1	ns
**Q15**	**How does your dedicated IBD nurse provide education on self-administration of subcutaneous medications to patients?**	7.3 ± 2.9	8.5 ± 1.9		5.1 ± 3.1	7.9 ± 2.6	8.3 ± 2.2	6.2 ± 3.2	
**Q16**	**How do you perceive your IBD nurse as a reference figure for the patients in your clinic?**	7.4 ± 2.9	8.6 ± 1.8		5.2 ± 3.2	7.7 ± 2.7	8.5 ± 2.3	6.0 ± 3.0	

The corrected Table 2 reads:

The ‘Fundamental Skills’ section associated with Table 2 has also been corrected to read:

“The analysis (Table 2) showed that patients who were able to identify their dedicated IBD nurse reported significantly higher scores in several key domains, including competence in providing information (8.4 ± 1.8 vs. 4.9 ± 3.1; p = 0.001), awareness of the physical impact of the disease (8.4 ± 1.8 vs. 4.9 ± 3.1; p = 0.0001), ability to establish an empathetic relationship (8.7 ± 1.7 vs. 5.2 ± 3.1; p = 0.001), and competence in managing biological therapies (8.7 ± 1.7 vs. 5.2 ± 3.1; p = 0.001). Across all other domains, although not statistically significant, a consistent trend toward higher scores was observed among patients who recognised their nurse. No statistically significant differences were found according to therapy type, although descriptively higher scores were observed among patients receiving intravenous therapies (Figure 2)”.

Instead of:

“The analysis revealed significant differences in patient perceptions depending on whether they could recognize their dedicated IBD nurse and the type of therapy they were receiving (Table 2). Patients who could identify their nurse reported notably higher satisfaction across various areas, including empathy, communication, and the nurse’s impact on their treatment journey. Accordingly, those who recognized their nurse rated the nurse’s empathy and support much higher (8.7 ± 1.7 vs. 5.2 ± 3.1), with a significant P-value of .001. They also reported a greater influence on their care (7.69 ± 2.28 vs. 3.88 ± 3.04), with a P-value of .0001. In terms of competence, patients who recognized their nurse felt more assured in the nurse’s capacity to manage their illness, with a mean score of 8.4 ± 1.8 compared to 4.9 ± 3.1, yielding a P-value of.0001. As for therapy, patients undergoing subcutaneous treatment reported significantly improved perceptions of their nurse’s competence and the impact on their care. They evaluated the nurse’s knowledge and ability to provide education higher (8.6 ± 2.1 compared to 6.0 ± 3.2) with a P-value of .02, and also indicated a greater impact on their care (7.9 ± 2.4 compared to 6.0 ± 3.1) with a P-value of .01 (Figure 2)”.

These corrections do not affect the findings or conclusions of the study.

