# Supplementary material for: The Dedicated Inflammatory Bowel Disease Nurse, If You Know Them, You Love Them: Survey of the Italian IBD Patients’ Association
Source: Crohns Colitis 360. 2025 Nov 11;7(4):otaf063. doi: 10.1093/crocol/otaf063 (PMC12659860; doi:10.1093/crocol/otaf063)
Supplement: otaf063_Supplementary_Data [file otaf063_supplementary_data.docx]

**Supplementary file**

Questionnaire

1. Region of residence
2. Age
3. Gender
   - Male
   - Female
   - Prefer not to answer
4. Diagnosis
   - Crohn’s Disease
   - Ulcerative Colitis
   - Indeterminate Colitis
5. Are you undergoing biological therapy?
   - Yes, subcutaneous biological therapy
   - Yes, intravenous biological therapy
   - No
6. In which year were you diagnosed?
7. Are you being followed by a specialized IBD center?
   - Yes
   - No
8. How many years have you been treated at the center currently following you?
9. Can you identify the dedicated nurse at your treatment center?
   - Yes
   - No
10. What kind of relationship do you have with your dedicated nurse?

- Excellent – I feel supported and can rely on them for any need.
- Good – The relationship is positive, but there is room for improvement.
- Neutral – I have no particular interactions, or the relationship is indifferent.
- Unsatisfactory – I do not feel adequately supported or do not receive enough attention.

1. To what extent has the presence of your dedicated nurse influenced your diagnostic/therapeutic journey?

- Rate from 1 to 10, where 1 represents "No influence" and 10 represents "Decisive influence".

1. In case of need, how much do you think your dedicated nurse can support you in solving your needs?

- Rate from 1 to 10, where 1 represents "No support" and 10 represents "Total support".

1. How do you assess your dedicated nurse’s competence in providing you with information about your disease?

- Rate from 1 to 10, where 1 represents "Not competent at all" and 10 represents "Extremely competent".

1. How well do you think the dedicated IBD nurse at your clinic knows the differences between IBD diagnoses and therapeutic pathways?

- Rate from 1 to 10.

1. How well does your dedicated IBD nurse understand the physical impact of your disease on you?

- Rate from 1 to 10.

1. How well does your dedicated IBD nurse understand the emotional and psychological impact of your disease on you?

- Rate from 1 to 10.

1. How competent is your dedicated IBD nurse in identifying needs and ensuring appropriate access to the best care for patients?

- Rate from 1 to 10.

1. How well does your dedicated IBD nurse establish an empathetic relationship with patients?

- Rate from 1 to 10.

1. How well does your dedicated IBD nurse support communication with the multidisciplinary team?

- Rate from 1 to 10.

1. How competent is your dedicated IBD nurse in managing perianal disease?

- Rate from 1 to 10.

1. How well can your dedicated IBD nurse manage a stoma or provide recommendations on specialists to consult?

- Rate from 1 to 10.

1. How well does your dedicated IBD nurse understand dietary issues related to your disease?

- Rate from 1 to 10.

1. How competent is your dedicated IBD nurse regarding the impact of incontinence on patients’ quality of life?

- Rate from 1 to 10.

1. How competent is your dedicated IBD nurse in discussing sexual health aspects related to IBD and providing necessary advice?

- Rate from 1 to 10.

1. How competent is your dedicated IBD nurse in managing biological therapies?

- Rate from 1 to 10.

1. How well does your dedicated IBD nurse provide health education for patients to self-administer subcutaneous medications?

- Rate from 1 to 10.

1. To what extent do you consider the IBD nurse to be a key reference figure for patients at your clinic?

- Rate from 1 to 10.

1. How well does your dedicated IBD nurse recognize symptoms of fatigue and suggest useful measures?

- Rate from 1 to 10.

1. How much do you believe your dedicated IBD nurse contributes to the multidisciplinary team and clinical discussions on patients?

- Rate from 1 to 10.

1. Does your dedicated IBD nurse attend congresses and training courses in the IBD field?

- Yes
- No
- I don’t know

1. Does your dedicated IBD nurse offer telephone or email support for patients?

- Yes
- No

1. How well does your dedicated IBD nurse educate patients on living with the disease, including using informational materials?

- Rate from 1 to 10.

1. How well does your dedicated IBD nurse provide health education for patient caregivers?

- Rate from 1 to 10.

1. How well does your dedicated IBD nurse know about technological innovations useful for managing IBD?

- Rate from 1 to 10.

1. How competent is your dedicated IBD nurse regarding current regulations and supporting patients in protecting their rights?

- Rate from 1 to 10.

1. How well does your dedicated IBD nurse manage patients in pre- and post-pregnancy phases?

- Rate from 1 to 10.

1. How competent is your dedicated IBD nurse in informing patients about travel requirements and care options away from home?

- Rate from 1 to 10.

1. How well does your dedicated IBD nurse understand the vaccination needs of patients and provide relevant information?

- Rate from 1 to 10.

1. How well does your dedicated IBD nurse recognize comorbidities and extraintestinal manifestations related to your disease?

- Rate from 1 to 10.

1. How competent and experienced is your dedicated IBD nurse in research?

- Rate from 1 to 10.

1. Does your dedicated IBD nurse conduct clinical studies?

- Yes
- No
- I don’t know

1. How well does your dedicated IBD nurse collaborate with colleagues from other specialties to provide multidisciplinary care?

- Rate from 1 to 10.

1. How competent is your dedicated IBD nurse in pain management?

- Rate from 1 to 10.
